# High Dose ESAs Are Associated with High iPTH Levels in Hemodialysis Patients with End-Stage Kidney Disease: A Retrospective Analysis

**DOI:** 10.3389/fpubh.2015.00258

**Published:** 2015-11-18

**Authors:** Lan Chen, Yi-Sheng Ling, Chun-Hua Lin, Jin-Xuan He, Tian-Jun Guan

**Affiliations:** ^1^Department of Nephrology, Zhongshan Hospital, Xiamen University, Xiamen, China

**Keywords:** erythropoiesis-stimulating agent, chronic kidney disease, hemodialysis, hemoglobin, parathyroid hormone

## Abstract

**Objective:**

Anemia and secondary hyperparathyroidism are the two most common complications associated with chronic kidney disease. Erythropoiesis-stimulating agents (ESAs) are widely used in the management of anemia in hemodialysis patients. A reverse correlation has been established between hyperparathyroidism and hemoglobin levels. The aim of this retrospective study is to evaluate the relationship of high-dose ESAs and hyperparathyroidism in hemodialysis patients with anemia.

**Methods:**

A total of 240 uremic patients maintained on regular hemodialysis were enrolled in this study. Among them, 142 patients were treated with Epiao^®^ (epoetin-alfa) and 98 patients were treated with Recormon^®^ (epoetin-beta). The target hemoglobin concentration was 110–130 g/L. Laboratory measurements including hemoglobin, calcium, phosphorus, albumin, intact-parathyroid hormone (iPTH), serum ferritin, and transferrin saturation were collected.

**Results:**

Hemoglobin concentration increased as iPTH level decreased by stratification. However, no significant association between anemia and calcium or phosphorus level was found. Patients with iPTH levels within 150–300 pg/mL had the highest levels of hemoglobin, serum ferritin, and transferrin saturation. Patients treated with Recormon and Epiao had similar hemoglobin concentrations. However, the dose of Recormon for anemia treatment was significantly less than that the dose of Epiao (*P* < 0.05). The level of iPTH in the Recormon group was significantly lower than in the Epiao group. In patients with hemoglobin levels between 110 and 130 g/L (*P* < 0.05), iPTH level was found to be significantly lower in patients treated with lower doses of ESAs than in patients treated with higher doses of ESAs, no matter which ESA was used (Recormon or Epiao, *P* < 0.05).

**Conclusion:**

The dose of ESAs might be positively associated with iPTH level, suggesting that a reasonable hemoglobin target can be achieved by using the lowest possible ESA dose.

## Introduction

Chronic kidney disease (CKD) is highly prevalent worldwide. In China, nearly 1 in 10 people have some degree of kidney dysfunction, totaling to almost 150 million patients (1). Anemia and secondary hyperparathyroidism are the two most common complications associated with CKD (2, 3). In fact, it has been reported that nearly 90% of patients with advanced CKD (classified as stage 4 and 5 CKD) suffer from anemia (4). The onset and severity of anemia has been shown to be well correlated with the decline in glomerular filtration rate (4). Early identification, evaluation, and treatment of anemia may decrease morbidity and mortality, as well as improve the quality of life, in CKD patients.

Secondary hyperparathyroidism is characterized by elevated serum parathyroid hormone (PTH) levels. Excess PTH may impair erythropoiesis by exerting a direct toxic effect on erythroid progenitor cells and an indirect effect through bone marrow fibrosis induction (5, 6). In addition, low hemoglobin concentration in uremic patients may have resulted from an increase in erythrocyte osmotic fragility due to high PTH concentration (7). A reverse correlation has been established between PTH and hemoglobin levels (8–10). Meytes et al. found that PTH concentrations within 7.5–30 U/mL, which is comparable to serum PTH levels in uremic patients, induced the significant inhibition of burst forming unit-erythroid (BFU-E) growth in murine bone marrow cultures, suggesting a possible pathway for the involvement of excess PTH in the genesis of the anemia of uremia (11).

Erythropoiesis-stimulating agents (ESAs) have become a hallmark of anemia therapy in patients with CKD and are the most commonly prescribed medication in dialysis patients, with >95% utilization in China (1). The first recombinant human erythropoietin, epoetin-alfa, was licensed in the United States in 1988 (12). Epoetin-alfa and Epoetin-beta are short-acting and cost-effective therapeutic agents and are administered — one to three times weekly in majority of CKD patients. However, its dosage increases with an extended dosing interval to achieve or maintain a target hemoglobin level. Epiao^®^(3,000 μ/tube) is a kind of Epoetin-alfa widely used in China, while Recormon^®^(2,000 μ/tube) is a kind of Epoetin-beta more commonly used in Europe, and has been used in China for approximately 4 years.

Findings from recent clinical trials have shown that doses that achieve high hemoglobin targets raised concerns of an increased risk of death or adverse outcomes such as thromboembolic events and stroke (13–16). Andrews et al. found that an increased dose frequency at a high-dose level increased the incidence of thrombotic toxicities compared to animals dosed less frequently in lower dose groups, despite a similarly high hematocrit across all groups (17), suggesting that a high hematocrit is not the sole causal factor leading to ESA-related toxicities, but it is also associated with dose level, dose frequency, and dosing duration. Thus, ESA-related toxicities independent of high hematocrit should be given close attention.

At present, limited data are available regarding the possible relationship between the dose of ESAs and PTH in uremic patients maintained on regular hemodialysis. This retrospective study aimed to investigate the association between the dose of ESAs and intact-PTH (iPTH) levels in hemodialysis patients based on clinical data in our hospital computer database system.

## Materials and Methods

### Patients and Procedures

This retrospective cohort study was conducted from February 2015 to May 2015 in Xiamen Zhongshan Hospital, Xiamen University, China. This study included uremic patients on maintained hemodialysis for at least 3 months, who used ESAs for the treatment of anemia. Patients who met the following criteria were excluded from the study: (1) age <18 years; (2) patients with active infection, malignancy, iron deficiency anemia (defined as ferritin <100 ng/mL and saturation <20%), or active bleeding within 3 months; (3) patients with incomplete data. Eligible patients were categorized into two groups, based on the ESA used: Recormon group and Epiao group.

### Data Collection

Data were obtained from Jinshida, the computer database system of Xiamen Zhongshan Hospital, which recoded the clinical data of 376 hemodialysis patients. The following data were collected: age, gender, dry weight, diagnosis of primary diseases, blood urea nitrogen (BUN), serum creatinine (Scr), hemoglobin, calcium, phosphorus, albumin, iPTH, serum ferritin, transferrin saturation, and dosage of ESAs. These data were measured in the morning on the day of hemodialysis (before dialysis). In accordance with Kidney Disease Improving Global Outcomes (KDIGO) guidelines, normal hemoglobin level was defined as levels between 110 and 130 g/L in uremic patients with maintained hemodialysis (8). Reference values for serum calcium and phosphorus were defined as values within 2.1–2.54 and 1.1–1.78 mmol/L, respectively (18). Patients were further classified based on iPTH levels: 150, 300, 600, and 1,500 pg/mL (19, 20).

### Study Ethics

This study was carried out by analyzing the retrospective data obtained from the Electronic Patient Record System of our hospital. The study protocol on human research was approved by the Ethics Committee of Zhongshan Hospital, Xiamen University. Written informed consent was obtained from all subjects.

### Statistical Analysis

Statistical analysis was performed using SPSS for Windows software version 17.0. Data were expressed as percentages or mean ± Standard Error of Mean (SEM). Demographic data and laboratory measurements among groups were compared by Pearson’s chi-squared test, Fisher’s exact-test, or Yate’s correction for continuity. A *P* value <0.05 was considered statistically significant.

## Results

### Characteristics of Uremic Patients Maintained on Regular Hemodialysis

A total of 240 uremic patients maintained on regular hemodialysis were included in this study. Baseline characteristics of patients are shown in Table 1. There were no significant differences in age, gender, dry weight, primary diseases, BUN, Scr, hemoglobin, serum phosphate, calcium, albumin, serum ferritin, and transferrin saturation between the Recormon and Epiao groups (Table 1, *P* > 0.05).

**Table 1:** **Baseline characteristics of patients**.

Variable	All subjects (*N* = 240)	ESAs treatment		*P* value
		Epiao^®^ (*n* = 142)	Recormon^®^ (*n* = 98)	
Age, years	57.85 ± 0.78	57.62 ± 0.94	58.47 ± 1.40	0.61
Gender, female, *n* (%)	92 (38.3)	57 (40.1)	35 (35.7)	0.08
Primary diseases, *n* (%)				0.99
Glomerular disease	90 (37.5)	52 (36.6)	38 (38.8)	
Diabetes mellitus	64 (26.7)	38 (26.8)	26 (26.5)	
Hypertension	36 (15.0)	21 (14.8)	15 (15.3)	
Lupus nephritis	10 (4.2)	6 (4.2)	4 (4.1)	
Autosomal dominant polycystic kidney disease	9 (3.7)	5 (3.5)	4 (4.1)	
Gouty nephropathy	8 (3.3)	5 (3.5)	3 (3.1)	
Vasculitis with renal damage	5 (2.0)	3 (2.1)	2 (2.0)	
Obstructive nephropathy	4 (1.7)	3 (2.1)	1 (1.0)	
Chronic transplant nephropathy	4 (1.7)	3 (2.1)	1 (1.0)	
Malignant tumor-associated renal injury	3 (1.3)	2 (1.4)	1 (1.0)	
Abercrombie degeneration	2 (0.8)	1 (0.7)	1 (1.0)	
Unknown reasons	5 (2.1)	3 (2.1)	2 (2.0)	
BUN (mmol/L)	23.97 ± 0.44	23.9 ± 0.56	24.08 ± 0.69	0.84
Scr (μmol/L)	990.34 ± 21.02	997.42 ± 26.68	980.08 ± 34.12	0.69
iPTH (pg/mL)	451.65 ± 26.94	531.05 ± 39.57	336.66 ± 57.76	<0.001
Calcium (mmol/L)	2.28 ± 0.01	2.29 ± 0.01	2.27 ± 0.47	0.47
Phosphorus (mmol/L)	2.01 ± 0.04	2.06 ± 0.04	1.94 ± 0.10	0.13
Albumin (g/L)	40.05 ± 0.27	40.2 ± 0.35	39.5 ± 0.44	0.51
Ferritin (ng/mL)	321.21 ± 14.62	298.84 ± 15.72	353.62 ± 27.4	0.08
Transferrin saturation (%)	31.74 ± 0.85	30.61 ± 1.12	33.38 ± 1.28	0.11
Hemoglobin (g/L)	103.15 ± 1.07	102.59 ± 1.38	103.54 ± 1.72	0.53
Dry weight (kg)	59.94 ± 0.60	59.05 ± 0.81	61.22 ± 0.86	0.07
ESAs total dose (μ/week)	5,781.25 ± 219.59	7,083.33 ± 264.93	3, 853.66 ± 225.13	<0.001
ESAs dose (μ/kg)	100.25 ± 4.15	124.41 ± 5.67	65.25 ± 3.82	<0.001
ESAs price/week (US$)	24.75 ± 1.34	12.99 ± 0.54	41.79 ± 2.26	<0.001

*Categorical variables are presented as percentages; continuous variables are presented as mean ± SEM. Demographic data and laboratory measurements among groups were compared by Pearson’s chi-squared test, Fisher’s exact-test, or Yate’s correction for continuity. The price of Epiao^®^ (epoetin-alfa): US$5.5/3,000 μ; the price of Recormon^®^ (epoetin-beta): US$21.5/2,000 μ*.

The average hemoglobin level of all subjects was 103.15 ± 1.07 g/L. Patients with a hemoglobin levels between 110 and 130 g/L accounted for 30.4% (*n* = 84). Anemia was divided into five classes based on hemoglobin level, according to the severity of anemia (21, 22): hemoglobin level <60 g/L (2.1%, *n* = 5), hemoglobin level ≥60 and <90 g/L (21.7%, *n* = 52), hemoglobin level ≥90 and <110 g/L (38.7%, *n* = 93), hemoglobin level ≥110 and <130 g/L (30.4%, *n* = 73), and hemoglobin level ≥130 g/L (7.1%, *n* = 17).

### Relationship between the Degree of Anemia and iPTH

**Figure 1:**
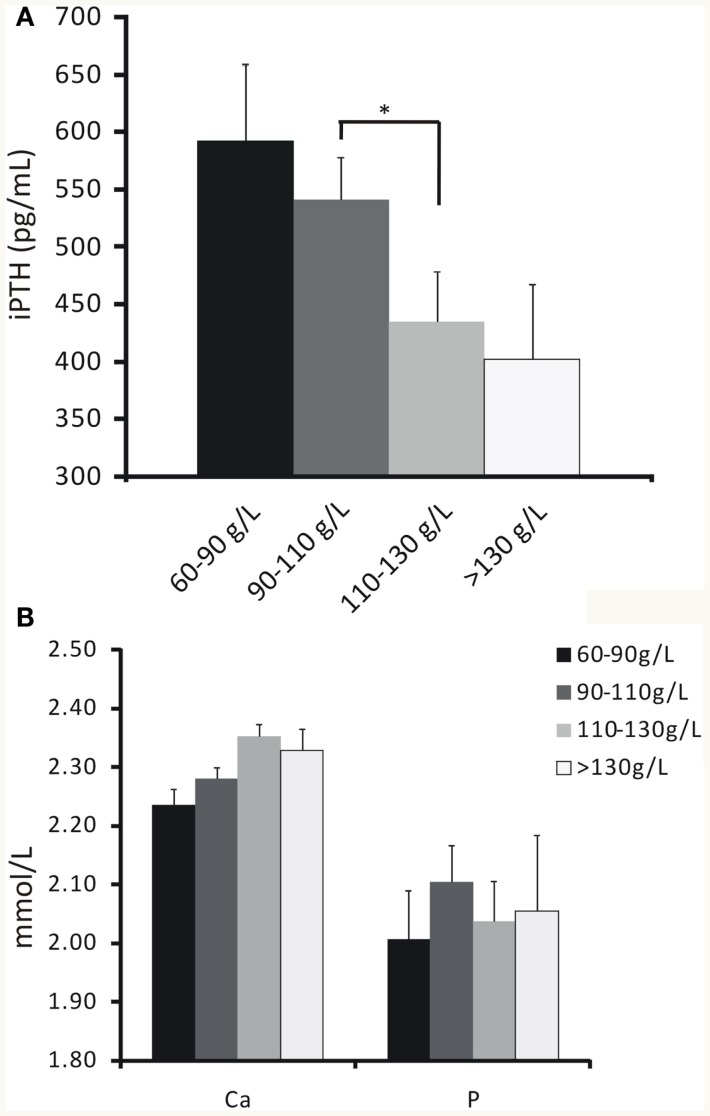
**The relationship between hemoglobin concentrations and iPTH, serum calcium or phosphorus levels**. **(A)** Hemoglobin concentration increased as iPTH level decreased by stratification. **(B)** No significant association between hemoglobin concentration and serum calcium or phosphorus level was found. Data were calculated by *t*-test. *P < 0.05.

The relationship between different degrees of anemia and iPTH was analyzed. Results revealed that hemoglobin level was negatively associated with iPTH (Figure 1A; Table 2; *P* < 0.05). However, no significant association between anemia and serum calcium or phosphorus level was found (Figure 1B; Table 2; *P* > 0.05 for both).

**Table 2:** **Measurements of serum calcium, phosphorus and iPTH levels based on hemoglobin values by stratification**.

Hb (g/L)	<60 (*n* = 5)	60–90 (*n* = 52)	90–110 (*n* = 93)	110–130 (*n* = 73)	>130 (*n* = 17)	*P* value
Ca (mmol/L)	–	2.23 ± 0.03	2.28 ± 0.02	2.34 ± 0.02	2.32 ± 0.04	
P (mmol/L)	–	2.06 ± 0.09	2.11 ± 0.06	2.04 ± 0.07	2.03 ± 0.12	
iPTH (pg/mL)	–	596.41 ± 86.17	506.81 ± 49.79	435.62 ± 69.56*	388.59 ± 83.59	

No statistically significant differences were found among the groups, except *P = 0.042 vs. patients with Hb within 90–110 g/L.

### Patients with 150–300 pg/mL of iPTH Had the Highest Levels of Hemoglobin, Serum Ferritin, and Transferrin Saturation

**Figure 2:**
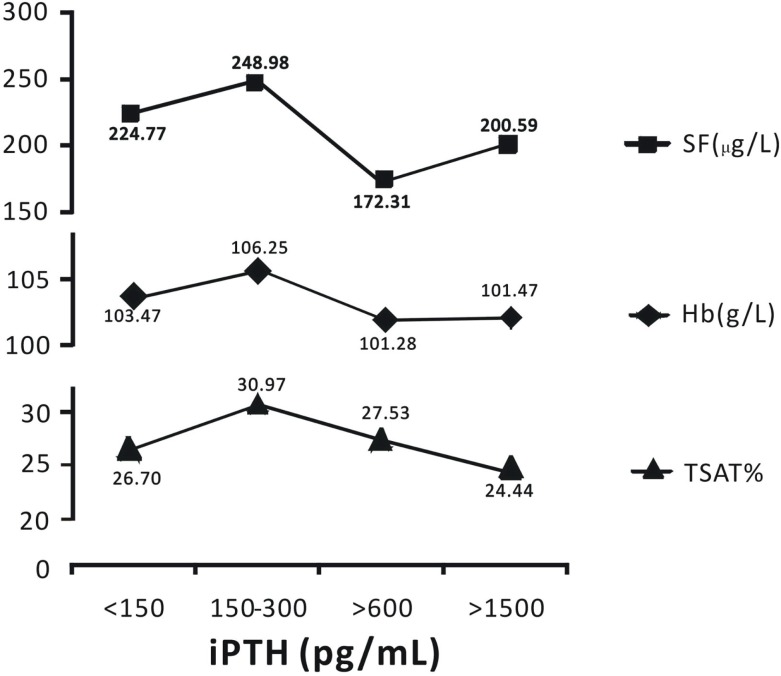
**Patients with iPTH levels within 150–300 pg/mL had the highest levels of hemoglobin, serum ferritin, and transferrin saturation**. Data are expressed as percentages or mean ± SEM.

When iPTH level was within 150–300 pg/mL, hemodialysis patients had the highest average levels of hemoglobin, serum ferritin, and transferrin saturation; which were 106.25 g/L, 248.98 μg/L, and 30.97%, respectively (Figure 2).

### Comparison of Recormon vs. Epiao in the Treatment of Anemia

**Figure 3:**
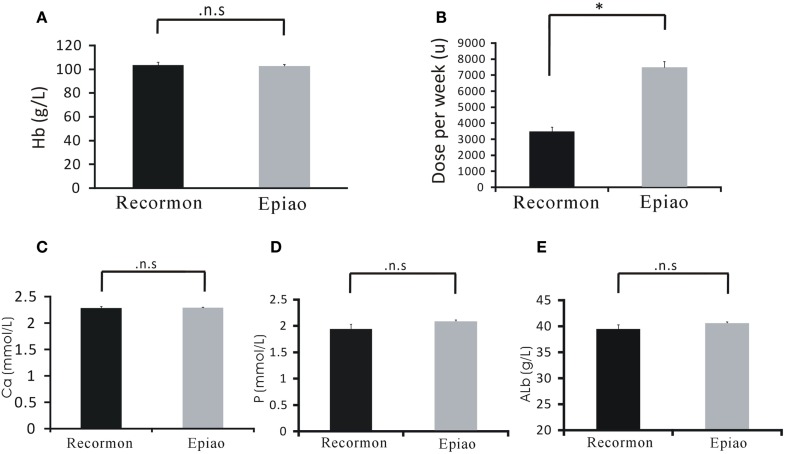
**Comparison of Recormon vs. Epiao in the treatment of anemia**. **(A)** Patients treated with Recormon and Epiao had similar hemoglobin levels (103.54 ± 1.72 g/L, *n* = 98 vs. 102.59 ± 1.38 g/L, *n* = 142, *P* > 0.05). **(B)** The dosage of Recormon for anemia treatment was significantly less than Epiao, 3,853.66 ± 225.13 μ/week, *n* = 98 vs. 7,083.33 ± 264.93 μ/week, *n* = 142. **(C–E)** There were no significant differences in calcium **(C)**, phosphorus **(D)**, and albumin **(E)** levels between the Recormon and Epiao groups. Data were calculated by t-test; *P* > 0.05.

Patients treated with Recormon and Epiao had similar hemoglobin levels (103.54 ± 1.72 g/L, *n* = 98 vs. 102.59 ± 1.38 g/L, *n* = 142, *P* > 0.05; Figure 3A). However, the dosage of Recormon (3,853.66 ± 225.13 μ/week, *n* = 98) for anemia treatment was significantly less than Epiao (7,083.33 ± 264.93 μ/week, *n* = 142, *P* < 0.05; Figure 3B, Table 1).

Calcium, phosphorus, and albumin serum levels were not significantly different between the Recormon and Epiao groups (respectively, 2.27 ± 0.47 vs. 2.29 ± 0.01 mmol/L, *P* > 0.05; 1.94 ± 0.10 vs. 2.06 ± 0.04 mmol/L, *P* > 0.05; 39.5 ± 0.44 vs. 40.2 ± 0.35 g/L, *P* > 0.05; Figures 3C–E; Table 1).

### The Relationship Between the Doses of ESAs and iPTH Levels

**Figure 4:**
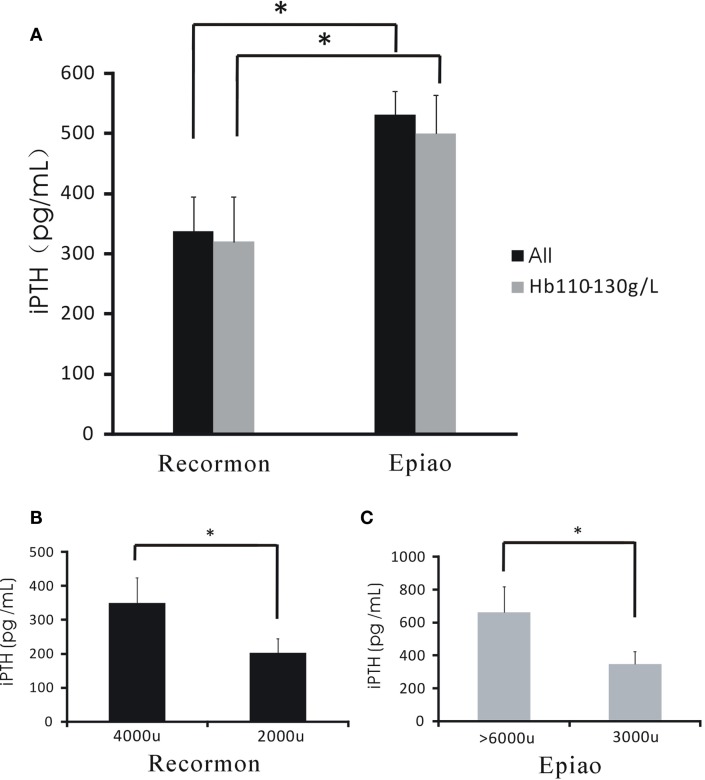
**The association between the dose of ESAs and iPTH levels**. **(A)** The level of iPTH in the Recormon and Epiao groups. **(B)** In the Recormon group, iPTH levels in patients treated with Recormon at 2,000 vs. 4,000 μ/week. **(C)** In the Epiao group, iPTH levels in patients treated with Epiao at 3,000 and >6,000 μ/week. Data were calculated by *t*-test; *P < 0.05.

For all hemodialysis patients, iPTH levels in the Recormon group was lower than the Epiao group (336.66 ± 57.76 pg/mL, *n* = 98 vs. 531.05 ± 39.57 pg/mL, *n* = 142; *P* < 0.05; Figure 4A; Table 1). A similar trend was found in hemodialysis patients with hemoglobin levels between 110 and 130 g/L (319.50 ± 74.9 pg/mL in the Recormon group, *n* = 28 vs. 499.5 ± 64.38 pg/mL in the Epiao group, *n* = 45; *P* < 0.05; Figure 4A).

Among patients with hemoglobin levels between 110 and 130 g/L, iPTH level was 201.54 ± 42.67 pg/mL (*n* = 10) in patients treated with Recormon at 2,000 μ/week, which was significantly lower than patients treated with Recormon at 4,000 μ/week (348.16 ± 117.9 pg/mL, *n* = 13; *P* < 0.05, Figure 4B). Similarly, average iPTH level in patients treated with Epiao at 3,000 μ/week was significantly lower than patients treated with Epiao >6,000 μ/week (347.15 ± 75.94 pg/mL, *n* = 12 vs. 661.01 ± 198.45 pg/mL, *n* = 27; *P* < 0.05, Figure 4C).

## Discussion

This retrospective study is the first to provide evidence that higher doses of ESAs (Epoetin-alfa and -beta) might be associated with higher levels of iPTH in uremic patients maintained on regular hemodialysis. Furthermore, we found that patients with iPTH levels within 150–300 pg/mL had the highest levels of hemoglobin, serum ferritin, and transferrin saturation, which was consistent with the recommended level of iPTH based on KDIGO guidelines (9).

Anemia is one of the most common complications of CKD. The World Health Organization has defined anemia as having a hemoglobin concentration lower than 13.0 g/dl in men and post-menopausal women, or a hemoglobin concentration <12.0 g/dL in other women. The use of ESAs markedly improved the lives of many anemic patients with CKD. However, high doses of ESAs have been shown to be associated with increased risk of adverse outcomes in adults and children with CKD. Patients treated with high ESA doses have a 1.2–1.5 increased risk of mortality (23). In this study, we found a significant reverse association between iPTH levels and hemoglobin concentrations in hemodialysis patients treated with ESAs, which is consistent with findings from previous studies (8–10).

In this study, patients in the Recormon and Epiao groups presented similar hemoglobin concentrations; however, the dosage used for Recormon was significantly lower than Epiao. We also found that iPTH level in the Recormon group was significantly lower than in the Epiao group. To further determine the association between the dose of ESAs and iPTH levels, we analyzed the clinical data of patients treated with Recormon or Epiao separately. The iPTH level was remarkably lower in patients treated with Recormon at 2,000 μ/week than patients treated with Recormon at 4,000 μ/week. A similar trend was found in patients treated with Epiao at a dosage of 3,000 or 6,000 μ/week, suggesting that high doses of ESAs may be related to high iPTH levels. These findings indicate that ESA toxicities could not be solely explained by high hematocrit. Given that high doses of ESAs may be associated to high levels of iPTH in maintained dialysis patients, patients with end-stage renal disease may best be managed by minimizing or withholding the dose of ESAs, especially in patients with recent cardiovascular or cerebrovascular events, hypertensive emergencies, or acute thromboembolic events. In fact, considering the increased risk for cardiovascular events at nearly normal hemoglobin concentrations and high doses of ESAs in CKD, it is not recommended in Taiwan to use disproportionately high dosages of ESAs to achieve a hemoglobin level within 100–110 g/L (24).

Although the pathogenesis of anemia in CKD is multifactorial, the lack of erythropoietin is the main cause of anemia in CKD patients (25). Additional factors contributing to CKD-associated anemia include iron deficiency, anemia of inflammation, suppression of erythropoiesis, shortening of red blood cell survival by uremic toxins, and blood loss such as gastrointestinal hemorrhage. Since iron deficiency is also a common causal factor for anemia, intravenous iron supplementation was encouraged earlier in Taiwan in 1996. Based on the experience of CKD anemia management in Taiwan, a reasonable hemoglobin target can be achieved by using the lowest possible ESA dose and intravenous iron supplementation (24).

Our study has several limitations. First, this observational study is a single center study. Second, given the potential confounding and selection bias by indication, cautious interpretation of these data is needed. Indeed, our observation on the association between iPTH and ESAs require further exploration by longitudinal prospective studies. Third, there is a predominance in the number of the patients treated with Epiao (142 for Epiao vs. 98 for Rocormon), which is probably due to the relatively cheap price of Epiao in comparison with Recormon (Table 1). Fourth, pharmacological interventions such as antihypertensive agents might have resulted in transient fluctuations or even interference in iPTH or hemoglobin levels.

In conclusion, this study demonstrates that higher doses of ESAs might be associated with higher levels of iPTH in uremic patients maintained on regular hemodialysis. The involvement of higher doses of ESAs in the pathogenesis of secondary hyperparathyroidism remains unclear and may require further investigation. Our findings suggest that using the lowest possible ESA dose can minimize potential risks, while achieving a reasonable hemoglobin target.

## Author Contributions

The manuscript was reviewed and approved by all authors, and is not under consideration for publication elsewhere in a similar form and in any language.

## Conflict of Interest Statement

The authors declare that the research was conducted in the absence of any commercial or financial relationships that could be construed as a potential conflict of interest.

## Abbreviations

BUN, blood urea nitrogen; CKD, chronic kidney disease; ESA, erythropoiesis-stimulating agent; Hb, hemoglobin; iPTH, intact-parathyroid hormone; Scr, serum creatinine.

## References

[1] Zhang L Wang F Wang L Wang W Liu B Liu J Prevalence of chronic kidney disease in China: a cross-sectional survey. Lancet (2012) 379:815–22. 10.1016/S0140-6736(12)60033-6 22386035

[2] Martin KJ Gonzalez EA. Metabolic bone disease in chronic kidney disease. J Am Soc Nephrol (2007) 18:875–85. 10.1681/ASN.2006070771 17251386

[3] Rodriguez M Nemeth E Martin D. The calcium-sensing receptor: a key factor in the pathogenesis of secondary hyperparathyroidism. Am J Physiol Renal Physiol (2005) 288:F253–64. 10.1152/ajprenal.00302.2004 15507543

[4] Levin A Thompson CR Ethier J Carlisle EJ Tobe S Mendelssohn D Left ventricular mass index increase in early renal disease: impact of decline in hemoglobin. Am J Kidney Dis (1999) 34:125–34. 10.1016/S0272-6386(99)70118-6 10401026

[5] Kwack C Balakrishnan VS. Managing erythropoietin hyporesponsiveness. Semin Dial (2006) 19:146–51. 10.1111/j.1525-139X.2006.00141.x 16551293

[6] Gallieni M Corsi C Brancaccio D. Hyperparathyroidism and anemia in renal failure. Am J Nephrol (2000) 20:89–96. 10.1159/000013563 10773607

[7] Wu SG Jeng FR Wei SY Su CZ Chung TC Chang WJ Red blood cell osmotic fragility in chronically hemodialyzed patients. Nephron (1998) 78:28–32. 10.1159/000044878 9453400

[8] Baradaran A Nasri H Intensification of anaemia by secondary hyperparathyroidism in hemodialysis patients. Med J Islam Acad Sci (2001) 14(4):161–6.

[9] Sliem H Tawfik G Moustafa F Zaki H. Relationship of associated secondary hyperparathyroidism to serum fibroblast growth factor-23 in end stage renal disease: a case-control study. Indian J Endocrinol Metab (2011) 15:105–9. 10.4103/2230-8210.81939 21731867 PMC3124995

[10] Trovato GM Carpinteri G Spina S Squatrito G Catalano D Iannetti E Hyperparathyroidism, anaemia and erythropoietin: effects on systolic function of dialysis patients. Abstracts of 31 st Congress of European Renal Association/European dialysis ND Transplantation Association, September 5-8, 1999 Madrid. Nephrol Dial Transpl (1999) 14:190.

[11] Meytes D Bogin E Ma A Dukes PP Massry SG. Effect of parathyroid hormone on erythropoiesis. J Clin Invest (1981) 67:1263–9. 10.1172/JCI110154 7229028 PMC370692

[12] Eschbach JW Abdulhadi MH Browne JK Delano BG Downing MR Egrie JC Recombinant human erythropoietin in anemic patients with end-stage renal disease. Results of a phase III multicenter clinical trial. Ann Intern Med (1989) 111(12):992–1000. 10.7326/0003-4819-111-12-992 2688507

[13] Besarab A Bolton WK Browne JK Egrie JC Nissenson AR Okamoto DM The effects of normal as compared with low hematocrit values in patients with cardiac disease who are receiving hemodialysis and epoetin. N Engl J Med (1998) 339:584–90. 10.1056/NEJM199808273390903 9718377

[14] Singh AK. Should we keep hemoglobin levels as a viable outcome measure? Nephrol News Issues (2010) 24(15–6):18. 20364493

[15] Phrommintikul A Haas SJ Elsik M Krum H. Mortality and target haemoglobin concentrations in anaemic patients with chronic kidney disease treated with erythropoietin: a meta-analysis. Lancet (2007) 369:381–8. 10.1016/S0140-6736(07)60194-9 17276778

[16] Regidor D McClellan WM Kewalramani R Sharma A Bradbury BD. Changes in erythropoiesis-stimulating agent (ESA) dosing and haemoglobin levels in US non-dialysis chronic kidney disease patients between 2005 and 2009. Nephrol Dial Transplant (2011) 26:1583–91. 10.1093/ndt/gfq573 20861195

[17] Andrews DA Pyrah ITG Boren BM Tannehill-Gregg SH Lightfoot-Dunn RM. High hematocrit resulting from administration of erythropoiesis-stimulating agents is not fully predictive of mortality or toxicities in preclinical species. Toxicol Pathol (2014) 42:510–23. 10.1177/0192623313486317 23674390

[18] Kidney Disease: Improving Global Outcomes (KDIGO) CKD-MBD Work Group. KDIGO clinical practice guideline for the diagnosis, evaluation, prevention, and treatment of Chronic Kidney Disease-Mineral and Bone Disorder (CKD-MBD). Kidney Int Suppl (2009) 113:S1–130. 10.1038/ki.2009.188 19644521

[19] Tentori F Blayney MJ Albert JM Gillespie BW Kerr PG Bommer J Mortality risk for dialysis patients with different levels of serum calcium, phosphorus, and PTH: the Dialysis Outcomes and Practice Patterns Study (DOPPS). Am J Kidney Dis (2008) 52(3):519–30. 10.1053/j.ajkd.2008.03.020 18514987

[20] National Kidney Foundation. K/DOQI clinical practice guidelines for chronic kidney disease: evaluation, classification, and stratification. Am J Kidney Dis (2002) 39(2 Suppl 1):S1–266. 11904577

[21] Pisoni RL Bragg-Gresham JL Young EW Akizawa T Asano Y Locatelli F Anemia management and outcomes from 12 countries in the Dialysis Outcomes and Practice Patterns Study (DOPPS). Am J Kidney Dis (2004) 44(1):94–111. 10.1053/j.ajkd.2004.08.005 15211443

[22] Kidney Disease: Improving Global Outcomes (KDIGO) Anemia Work Group. KDIGO clinical practice guideline for anemia in chronic kidney disease. Kidney Int Suppl (2012) 2:279–335.

[23] Suttorp MM Hoekstra T Mittelman M Ott I Krediet RT Dekker FW Treatment with high dose of erythropoiesis-stimulating agents and mortality: analysis with a sequential Cox approach and a marginal structural model. Pharmacoepidemiol Drug Saf (2015) 24(10):1068–75. 10.1002/pds.3855 26265483

[24] Hung SC Kuo KL Tarng DC Hsu CC Wu MS Huang T-P. Anaemia management in patients with chronic kidney disease: Taiwan practice guidelines. Nephrology (Carlton) (2014) 19:735–9. 10.1111/nep.12332 25156587

[25] Randolph JF Scarlett JM Stokol T MacLeod JN Clinical effi-cacy and safety of recombinant canine erythropoietin in dogs with anemia of chronic renal failure and dogs with recombinant human erythropoietin-induced red cell aplasia. J Vet Intern Med (2004) 18:81–91. 10.1111/j.1939-1676.2004.tb00139.x 14765736

